# Acute Cytomegalovirus Infection Associated With Splenic Infarction: A Case Report and Review of the Literature

**DOI:** 10.7759/cureus.23404

**Published:** 2022-03-22

**Authors:** George A Blackwood, Mark Danta, Rohan Gett

**Affiliations:** 1 Northern Clinical School, The University of Sydney, Sydney, AUS; 2 Faculty of Medicine, University of New South Wales, St. Vincent's Hospital, Sydney, AUS; 3 General and Colorectal Surgery, University of New South Wales, St. Vincent's Hospital, Sydney, AUS

**Keywords:** cmv, ebv, splenic rupture, thrombosis, mononucleosis, splenic infarction, cytomegalovirus

## Abstract

Splenic infarction associated with acute cytomegalovirus infection (CMV) in immunocompetent patients was initially described as a very rare occurrence but has been reported in recent years with increasing frequency. Many cases undergo multiple investigations only to leave acute CMV as the likely cause. There is a risk of splenic rupture and, although this complication is rare, fatalities have occurred.

Although the exact mechanism of CMV as a vascular pathogen is unclear, there are now multiple reports describing venous thrombosis and arterial infarction in the presence of this acute viral infection. Our case prompted a review of the literature, and we suggest splenic infarction should be recognised as a possible complication of acute CMV.

## Introduction

The first known case of cytomegalovirus infection (CMV)-related splenic infarction was reported by Jordan et al. in 1973 in a 26-year old, previously healthy American woman [1]. Over 30 years later, in 2008, Atzmony et al. described two large splenic infarctions in a 36-year-old Caucasian woman, which the authors reported to be only the third known case worldwide of CMV-related splenic infarction in an immunocompetent patient [2].

Since these early days, cases have been reported with increasing frequency in the literature-Kassem et al. [3] in France, Shimizu et al. [4] in Japan, and Rawla et al. [5] in the USA, all reporting in 2017 splenic infarction with acute CMV in immunocompetent patients aged 32, 37, and 62 years, respectively. In 2019, another two cases were reported-Schattner et al. [6] in Israel, describing a healthy 34-year-old woman, and Redondo et al. [7] in Spain, reporting on a 63-year-old HIV-negative woman. A further case was reported by Pakkiyaretnam et al. in 2020 in England, describing another previously healthy 23-year-old female where meningitis was the initial suspected diagnosis but acute CMV with splenic infraction was subsequently confirmed [8].

## Case presentation

Our case was a 28-year-old male chef who presented with a four-day history of fevers, sweats, cough, headaches, myalgia, and left upper quadrant pain. He had previously been in good health and, on physical examination, he had tenderness in his left upper abdominal quadrant with a temperature of 38.1 °C, but his vital signs were otherwise normal. Initial blood tests showed a white blood cell count of 12.7 × 109/L (4.0-11.0) with a lymphocytosis of 8.4 × 10.9/L (1.5-4.0) and a monocytosis of 1.5 × 109/L (0.2-1.0). Liver function tests (LFTs) revealed elevated alanine transaminase (ALT) at 246 U/L (0-30), aspartate aminotransferase (AST) at 78 U/L (0-30), gamma glutamyl transaminase (GGT) at 82 U/L (0-35), and lactate dehydrogenase (LDH) at 656 U/L (0-430). Hepatitis A, B, and C were negative, as was Epstein-Barr virus (EBV) IgM, but with a positive IgG consistent with previous infection. CMV IgM was positive and CMV IgG was 8.6 AU/ml (ULN 6.0 AU/ml). CMV phosphoprotein 65 (pp65) was not measured.

**Figure 1:**
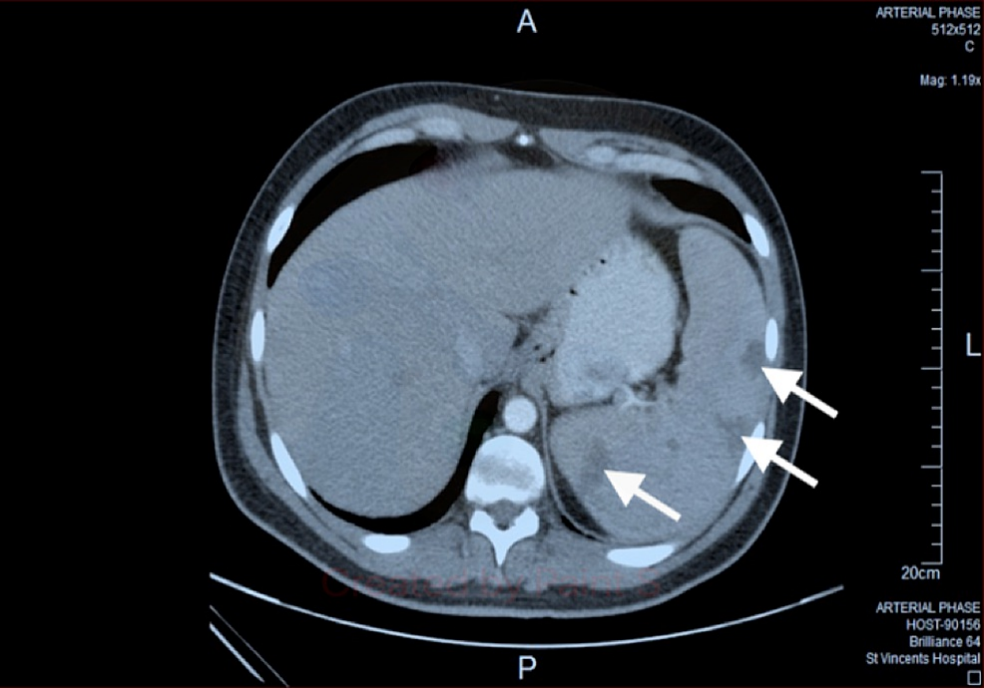
An abdominal CT scan performed eight days after admission showed splenomegaly with peripheral wedge shaped splenic infarcts.

He continued to report abdominal pain and underwent ultrasound, which showed splenomegaly with three hypoechoic focal abnormalities, and then underwent an abdominal CT scan, which demonstrated multiple wedge-shaped hypodensities. The CT did not identify thrombus in the splenic artery, but the scan was reported as consistent with splenic infarcts (Figure 1). A surgical opinion was obtained regarding the risk of rupture and the need for splenectomy, but he was treated conservatively. The patient proceeded to have more investigations, including a trans-thoracic echocardiogram, which was normal, and flow cytometry, showing no evidence of leukaemia or lymphoma. D-dimer was mildly elevated at 1.08 mg/L (ref <0.5 mg/L), a thrombophilia and autoimmune screen were unremarkable, HIV serology was negative, and no other pathogens were identified. The patient was not given anti-viral therapy but improved after two weeks and made a full recovery. Follow-up imaging did not occur, and repeat serology one month after presentation revealed that the CMV IgG had increased to 99.2 AU/ml.

## Discussion

Splenic infarction can occur in association with a variety of conditions, including haematological malignancies, hypercoaguable states, thromboembolic disorders, and trauma. It has also been reported with parasitic infections such as malaria and babesiosis [9], and also with acute EBV and CMV. Whilst these viral infections are both very common, EBV largely occurs in teenagers and young adults, whereas CMV infection increases with age, with serological evidence of exposure rising from 36% in 6-11-year olds to 91% of the population aged 80 years or older [10].

CMV has been reported in association with thrombosis. In 2010, Atzmony et al. studied 140 hospitalised patients with acute CMV matched to 140 consecutive controls and reported nine patients with thrombosis (6.4%) in the CMV group with no episodes of thrombosis in the control group. Five of these patients had arterial thrombosis (four splenic and one renal infarct), four had venous thrombosis, and the authors concluded that acute CMV is associated with thrombosis independently of other risk factors [11].

In 2010, Justo et al. conducted a meta-analysis on 97 cases with thrombosis associated with acute CMV, of which 64 were immunocompetent and 33 were immunocompromised. Although deep vein thrombosis/pulmonary embolism was the most common vascular complication with 52% of the total affected, splenic infarction occurred in 12 patients (12.4%), with 10 patients in the immunocompetent group and two patients who were immunocompromised. The authors concluded that there is a true need for a prospective study on hospitalised and ambulatory patients with thrombosis to be tested for recent CMV infection [12].

The pathophysiology of CMV-associated vascular complications is not fully understood. Westphal et al. reported in 2006 that CMV-DNA in smooth muscle cells induces local growth factor expression as well as endothelial activation and suggested that CMV plays a crucial role in mediating the progression of atherosclerosis [13]. In 2014, Protopapa et al. reported another case of CMV and splenic infarction and described several mechanisms for vasculopathy, including platelet and leucocyte adhesion to infected endothelial cells [14].

Irrespective of the mechanism, acute CMV infection appears to be a vascular pathogen. In our case, there was an initial reluctance to attribute the splenic lesions to CMV, which led to additional investigations for other causes. The issues in management are related to the role of antivirals for immunocompetent patients, which is not clear, and the possibility of splenic rupture. This complication appears to be rare in acute CMV, but it was reported in 2014 by Vidarsdottir et al. in a 53-year old woman who concluded that primary CMV infection can cause splenic rupture without a history of trauma in immunocompetent adults [15].

More commonly, splenic rupture has been reported in infectious mononucleosis (IM), though many of the early studies did not differentiate between EBV and CMV, both of which are known to cause IM [16]. Although a rare event, fatalities have occurred with acute splenic haemorrhage the most common cause of death in IM [17]. The risk of death from splenic rupture specifically associated with CMV is unknown, but a systematic review by Bartlett et al. of 85 cases of splenic rupture with IM reported a 9% mortality [18]. This 2016 review examined published cases between 1984 and 2014, and although this review did not identify the cause of mononucleosis, it is considered that CMV is more likely to be the cause of splenic rupture associated with IM rather than its more benign EBV relation.

## Conclusions

Previously rarely reported, splenic infarction associated with acute CMV has been described with increasing frequency in recent years. This unexpected complication causes diagnostic and management difficulties, with cases tending to undergo multiple investigations only to leave CMV as the likely pathogen and cause. There is a risk of splenic rupture, though it appears rare, and most cases can be managed conservatively. Whilst CMV can often present as a relatively mild infection, it appears to be pro-thrombogenic and questions remain over the role of screening for thrombosis and prophylactic anticoagulation. There are now multiple reports describing a similar clinical picture to our case, and we suggest that splenic infarction should be recognised as a possible complication in acute CMV infection.
